# Biomarkers and Strain Echocardiography for the Detection of Subclinical Cardiotoxicity in Breast Cancer Patients Receiving Anthracyclines

**DOI:** 10.3390/jpm13121710

**Published:** 2023-12-14

**Authors:** Aditi A. Bhagat, Andreas P. Kalogeropoulos, Lea Baer, Matthew Lacey, Smadar Kort, Hal A. Skopicki, Javed Butler, Michelle Weisfelner Bloom

**Affiliations:** 1 Division of Cardiology, Stony Brook University, Stony Brook, NY 11794, USA; aditi.bhagat@stonybrookmedicine.edu (A.A.B.); andreas.kalogeropoulos@stonybrookmedicine.edu (A.P.K.); smadar.kort@stonybrookmedicine.edu (S.K.); hal.skopicki@stonybrookmedicine.edu (H.A.S.); 2 Division of Oncology, Stony Brook University, Stony Brook, NY 11794, USA; lea.baer@stonybrookmedicine.edu; 3 Division of Cardiology, University of Michigan Medical Center, Ann Arbor, MI 48109, USA; mlacey@med.umich.edu; 4 Division of Cardiology, University of Mississippi Medical Center, Jackson, MS 39216, USA; jbutler4@umc.edu

**Keywords:** anthracycline cardiotoxicity, cardio-oncology, heart failure, biomarkers

## Abstract

The optimal surveillance and management strategies for breast cancer patients receiving anthracycline therapy are limited by our incomplete understanding of the role of biomarkers heralding the onset of cardiotoxicity. The purpose of this study was to determine whether there is a temporal correlation between cardiac biomarkers and subclinical left ventricular dysfunction in breast cancer patients receiving anthracycline chemotherapy. Thirty-one females between 46 and 55 years old with breast cancer treated with anthracycline chemotherapy were prospectively enrolled. Cardiac biomarkers were correlated with echocardiography with speckle tracking at baseline, post-anthracycline therapy, and 6 months post-anthracycline chemotherapy. Subclinical cardiotoxicity was defined as ≥ 10% reduction in global longitudinal strain (GLS). There was a relative reduction in left ventricular ejection fraction (LVEF) ≥ 10% in 5/30 (17%) and 7/27 (26%) patients post-anthracycline therapy and 6 months post-anthracycline therapy, respectively. Subclinical cardiotoxicity was noted in 8/30 (27%) and 10/26 (38%) patients post-anthracycline and 6 months post-anthracycline therapy, respectively. Baseline N-terminal pro B-type natriuretic peptide (NT-proBNP) was the strongest predictor of LVEF (ρ = −0.45; *p* = 0.019), with post-therapy NT-proBNP values illustrating similar predictive value (ρ = −0.40; *p* = 0.038). Interim changes in suppression of tumorigenicity 2 (ST2) and galectin-3 correlated with a 6-month change in LVEF (ρ = −0.48; *p* = 0.012 and ρ = −0.45; *p* = 0.018, for ST2 and galectin-3, respectively). Changes in galectin-3 from baseline to mid-therapy paralleled changes in GLS. NT-proBNP, ST2, and galectin-3 correlate with reduced LVEF among breast cancer patients receiving anthracycline therapy. Additional trials focusing on a cardiac biomarker approach may provide guidance in the early diagnosis and management of anthracycline-induced cardiotoxicity.

## 1. Introduction

Anthracycline-based chemotherapy, namely doxorubicin and daunorubicin, has been a cornerstone in breast cancer treatment for nearly fifty years [1], significantly reducing mortality by as much as 20–30% [2]. Despite the utilization of lower doses of anthracycline therapy to treat breast cancer patients relative to those used in other malignancies, anthracyclines are associated with both short- and long-term increases in cardiovascular events, including heart failure (HF) [3]. Chronic anthracycline-induced cardiotoxicity typically presents within one year of therapy and is often irreversible, significantly impacting overall survival and prognosis in breast cancer survivors [3]. Moreover, late-onset cardiotoxicity occurring up to 15 years after completion of anthracycline therapy may appear in multiple forms, including as a new onset arrhythmia, left ventricular dysfunction (LVD), or clinical HF. To date, no well-defined surveillance strategies have been developed to aid in the detection and management of cardiotoxicity among patients receiving anthracycline chemotherapy [4,5].

Echocardiographic imaging has become the mainstay of cardiac monitoring in cancer patients owing to its widespread availability, safety, and reliability [6]. However, the COVID-19 era, in particular, highlighted that data are lacking to guide the timing and frequency of imaging prior to and after anthracycline therapy [6]. Furthermore, among patients who do indeed develop clinical cardiotoxicity, guidelines for follow-up management remain unclear [6]. New techniques, including contrast-enhanced three-dimensional echocardiography, have high reproducibility and may increase the ability to detect even subclinical LVD [6,7], providing an advantage in cancer patients in whom subtle differences in cardiac function may have important implications [8]. Strain echocardiography can also be used to assess myocardial function in more detail, most commonly based on the displacement of myocardial speckles, which are patterns formed from natural acoustic markers in grey-scale ultrasound images (i.e., speckle tracking) [9]. In patients who develop HF, reduction in echocardiographic global longitudinal strain (GLS) precedes LVD [10]. However, newer techniques are needed to identify patients at elevated risk for subclinical cardiotoxicity as detected by strain imaging.

B-type natriuretic peptide (BNP) and N-terminal pro B-type natriuretic peptide (NT-proBNP) are biomarkers of myocardial stress that have predictive value in chemotherapy toxicity prediction, with persistent elevations portending worse prognosis [11,12]. Cardiac troponin I and cardiac troponin T, markers of myocardial cell injury or death, have high negative predictive value for cardiotoxicity [13,14,15]. Suppression of tumorigenicity 2 (ST2), a member of the interleukin 1 receptor family and a marker of cardiac inflammation, can be elevated in response to myocardial stressors such as myocardial infarction and acute HF, with elevated levels portending higher mortality [16]. Galectin-3, a marker of myocardial fibrosis, is involved in several biological processes, including inflammation, fibrosis, and immune response, which can be indicative of cardiac remodeling. Recently, elevations in galectin-3 were correlated with the development of anthracycline-induced cardiac toxicity in breast cancer patients [17], but there are limited data on the role of galectin-3 in the breast cancer population.

We conducted a small, prospective, pilot cohort study among breast cancer patients receiving anthracycline-based chemotherapy to evaluate the temporal changes in the levels of serum biomarkers NT-proBNP, ST2, high-sensitivity cardiac troponin I (hs-cTnI), and galectin-3 in relation to changes in subclinical LVD as assessed by two-dimensional echocardiography or three-dimensional echocardiography with strain imaging. Our secondary aims were to determine the most specific biomarker for prediction of subclinical cardiotoxicity, the frequency of alterations in chemotherapy regimens due to detection of subclinical cardiotoxicity, and the frequency of initiation or change in cardio-protective agents due to subclinical detection of cardiotoxicity as detected by echocardiography or biomarkers.

## 2. Materials and Methods

### 2.1. Overview

This was a prospective, pilot cohort study conducted among female subjects 18–85 years old with a diagnosis of biopsy-proven invasive breast cancer without metastases who were planned for anthracycline-inclusive chemotherapy and followed up for a minimum of 6 months after completion of treatment. Patients prospectively underwent serial blood and echocardiographic assessment at prespecified time points prior to, during, and after anthracycline therapy. The Institutional Review Board of Stony Brook University approved the study (protocol #922042). Informed consent was obtained from all participants. The study protocol and procedures conformed to the standards set by the latest revision of the Declaration of Helsinki.

### 2.2. Definition of Chemotherapy-Induced Cardiotoxicity

Cancer therapy-related cardiotoxicity has been defined by both the Food and Drug Administration and Cardiac Review and Evaluation Criteria. The Food and Drug Administration defines anthracycline cardiotoxicity as a decrease in left ventricular ejection fraction (LVEF) ≥ 20% when the baseline LVEF is normal or a decrease in LVEF ≥ 10% when the baseline LVEF is less than the institutional lower limit of normal [18]. The Cardiac Review and Evaluation Criteria supervising trastuzumab trials defines cardiotoxicity as a decrease in LVEF ≥ 5% (either global or more severe in the septum) with an absolute LVEF < 55% and accompanying symptoms of clinical HF, or a decrease in LVEF ≥10% with an absolute LVEF < 55% without clinical HF [18].

Subclinical cardiotoxicity is difficult to define with one universal GLS value owing to differences in echocardiographic machine vendors, strain software, and patient characteristics such as gender and age [8]. A systematic review found a change in GLS of 10–15% to be the most predictive parameter for the development of cardiotoxicity [19]. In patients receiving trastuzumab alone or in combination with anthracyclines, a change in GLS >11% remained a strong predictor of cardiotoxicity [20]. For the purposes of this study, given the data available at the time of initiation, we defined subclinical cardiotoxicity as ≥10% reduction in GLS [8].

### 2.3. Study Population

This was a single-center study conducted at an academic institution between the years of 2017 and 2020. Thirty-nine female subjects between the ages of 18 and 85 years old treated with anthracycline-inclusive chemotherapy for biopsy-proven invasive breast cancer were screened for this study. Exclusion criteria were as follows: (1) history of major heart disease at the time of breast cancer diagnosis (myocardial infarction or known LVD at baseline, defined as LVEF < 40%); (2) history of known obstructive coronary artery disease or coronary revascularization within the past 1 year; (3) history of clinical HF or previous HF hospitalization; (4) patients with elevations in NT-proBNP (within 2 times the upper limit of normal), ST2, galectin-3, or hs-cTnI above the upper limit of normal during baseline screening; (5) patients with metastatic disease or recurrent breast cancer at diagnosis; and (6) history of other chemotherapy treated malignancy.

### 2.4. Monitoring

Patients were recruited and followed for a period of at least 6 months. At the first visit, baseline information was obtained, including a medical history, physical exam, and patient demographics. Patients had laboratory evaluation performed at the baseline visit, mid-anthracycline therapy, post-anthracycline chemotherapy, and 6 months post-anthracycline chemotherapy. These labs included cardiac biomarkers (hs-cTnI, NT-proBNP, C-reactive protein CRP, galactin-3, and ST2). An electrocardiogram and echocardiogram with speckle tracking were performed at the baseline visit, post-anthracycline chemotherapy, and 6 months post-anthracycline chemotherapy. Seventy-seven transthoracic echocardiograms were obtained using the GE Healthcare Vivid 95 machine (GE HealthCare, Chicago, IL, USA), and ten echocardiograms were obtained with the GE Healthcare Vivid E9 machine (GE HealthCare, Horten, Norway). Strain parameters were obtained using GE EchoPac versions 113 and 202 (GE Healthcare, Horten, Norway), respectively. Both machines used the M5Sc transducers for two-dimensional imaging and strain imaging, and the 4 V probe was used for the calculation of three-dimensional LVEF and three-dimensional left ventricular volumes. All 88 echocardiograms had both two-dimensional and three-dimensional data available. Per the discretion of the reading cardiologist, three-dimensional generated LVEF was reported in 74/88 echocardiograms, while the remaining 14/88 echocardiograms had two-dimensional LVEF reported. GLS was calculated by averaging the values of peak systolic strain obtained using speckle imaging from 17 segments. All echocardiograms were read at a single primary academic center where the study was conducted.

### 2.5. Statistical Analysis

Continuous variables were described as a median (interquartile range) to ensure valid measures of location and dispersion regardless of distribution and frequency (percentage) to describe discrete (binary and categorical) variables. To examine changes over time for the variables of interest, we employed mixed-effects linear models with the patient as a random variable (i.e., each patient had an individual intercept for each variable of interest). Biomarkers were log-transformed before modeling because of log-normal distributions. Changes from the baseline for each variable of interest were assessed with prespecified contrasts for the marginal means after estimating the corresponding mixed-effects model. A joint contrast (i.e., overall difference from baseline) was used to assess the magnitude and significance of changes vs. baseline values. Linear correlations between changes were examined with the Spearman correlation coefficients. We used unadjusted α = 0.05 as the threshold for statistical significance. Analyses were performed with STATA 17.0 (StataCorp LLC, College Station, TX, USA).

## 3. Results

### 3.1. Study Population

A total of 39 patients were initially screened for this study. Six participants were excluded secondary to screen failures, including one patient with a baseline NT-proBNP twice the upper limit of normal, one patient with metastasis detected after enrollment, two patients with a chemotherapy regimen that was altered to no longer include anthracycline therapy, and two patients who failed to complete baseline laboratory bloodwork. Two patients withdrew from the study. The 31 patients included in the study were followed for 6 months post-anthracycline chemotherapy. The baseline characteristics for the 31 patients are presented in Table 1. The median age was 50 years old (ranging from 46 to 55). Approximately 6.5% of the patients were of Black descent, and 6.5% of the patients were of Hispanic descent. All 31 patients received dose-dense doxorubicin therapy and cyclophosphamide, while 29 patients also received paclitaxel. Four patients received trastuzumab therapy. The anthracycline dose for all patients was 60 mg/m^2^ (median cumulative dose of 106 mg), and the cyclophosphamide dose for all patients was 600 mg/m^2^ (median cumulative dose of 1060 mg). The median paclitaxel dose was 80 mg/m^2^, and the trastuzumab dose was 8 mg/m^2^ for all patients who received therapy.

### 3.2. Follow-Up

The follow-up assessments are summarized in Table 2. Twenty-seven of the thirty-one patients included were able to complete all follow-up biomarker, electrocardiogram, and echocardiography assessments at 6 months post-anthracycline therapy. The reason for missed assessments was principally due to the COVID-19 pandemic.

### 3.3. Imaging

Echocardiography findings at the baseline visit, post-therapy visit, and 6 months post-therapy visit are summarized in Table 3. The median LVEF was 63% at baseline and 60% both immediately post-anthracycline chemotherapy and 6 months post-anthracycline chemotherapy.

**Figure 1:**
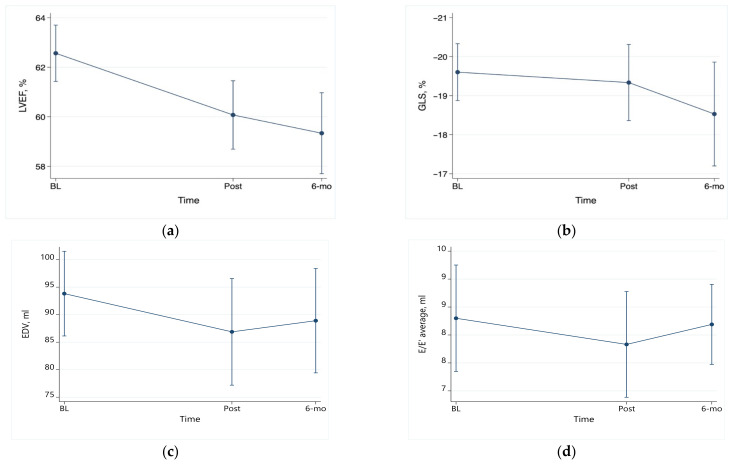
Serial changes in echocardiographic parameters. (**a**) LVEF: post-therapy vs. baseline: *p* < 0.05; 6 months vs. baseline: *p* < 0.005. (**b**) GLS: all changes nonsignificant; (**c**) EDV: all changes nonsignificant; (**d**) E/E’ average: all changes nonsignificant.

LVEF was noted to be significantly reduced at the post-therapy visit and the 6-month visit in comparison to baseline by 2.5% (95% confidence interval CI 0.8 to 4.4; *p* = 0.010) and 3.2% (95% CI 1.2 to 5.2; *p* = 0.002), respectively (Figure 1a). Of the 30 patients with repeat echocardiography assessment post-therapy, 5 (17%) had a relative reduction in LVEF ≥ 10%, and 1 had an absolute reduction in LVEF ≥ 10% (of note, the nadir of EF in this patient was 55% and subsequently increased to 63%). Seven of twenty-seven patients (26%) with repeat assessment 6 months after therapy had a relative reduction in LVEF ≥ 10%.

Changes in GLS were not statistically significant (*p* = 0.32 for the joint contrast vs. baseline). In comparison to the baseline GLS, there was no statistically significant decrease in the GLS 6 months post-chemotherapy (Figure 1b). However, of the 30 patients with repeat assessment post-therapy, 8 (27%) developed subclinical cardiotoxicity, which was defined as a relative reduction in GLS ≥ 10%. Similarly, 10 of the 26 patients (38%) with repeat assessment 6 months after therapy developed subclinical cardiotoxicity.

Left ventricular volumes did not change significantly over time (Figure 1c). While E velocities were lower immediately post-anthracycline therapy and remained so 6 months after therapy (*p* = 0.005 for the joint contrast vs. baseline), A velocities did not change (*p* = 0.52). Changes in the septal, lateral, and average E’ velocities were all statistically significant post-therapy and 6 months post-therapy vs. baseline (*p* = 0.007, *p* = 0.001, and *p* = 0.001, respectively). However, the E/E’ average ratio did not change significantly (Figure 1d).

### 3.4. Biomarkers

Table 4 summarizes the main biomarker findings at each visit. All troponin values were at the lower limit of detection (<0.01 mg/mL). Similarly, we did not find statistically significant differences in CRP over time.

**Figure 2:**
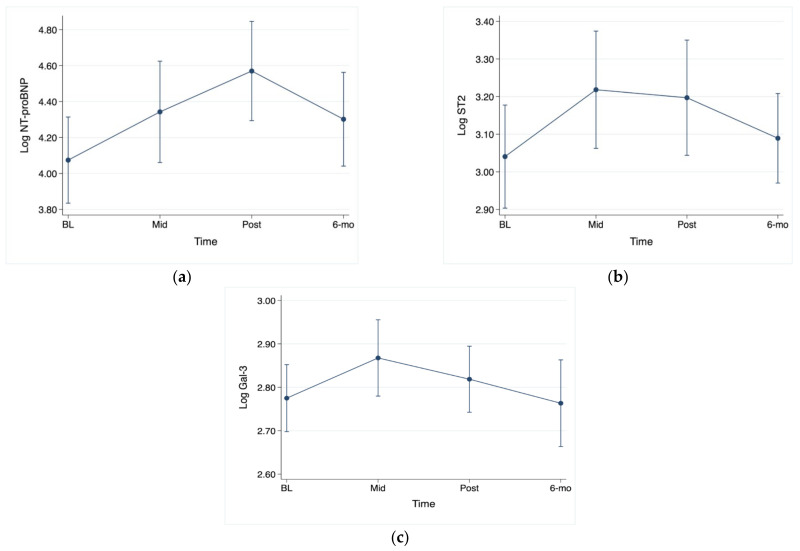
Serial changes in biomarkers. (**a**) NT-proBNP: post-therapy vs. baseline, *p* < 0.001; (**b**) ST2: mid-therapy vs. baseline, *p* < 0.05; post-therapy vs. baseline, *p* < 0.05; (**c**) galectin-3: mid-therapy vs. baseline, *p* < 0.05.

NT-proBNP was significantly increased at the post-chemotherapy visit in comparison to the baseline NT-proBNP (Figure 2a). ST2 has significantly increased at both the mid-therapy and post-therapy visits in comparison to baseline, with *p* < 0.05 for both comparisons (Figure 2b). Galectin-3 was significantly increased mid-therapy in comparison to baseline (Figure 2c).

### 3.5. Biomarkers as Predictors of Changes in LVEF and GLS

**Figure 3:**
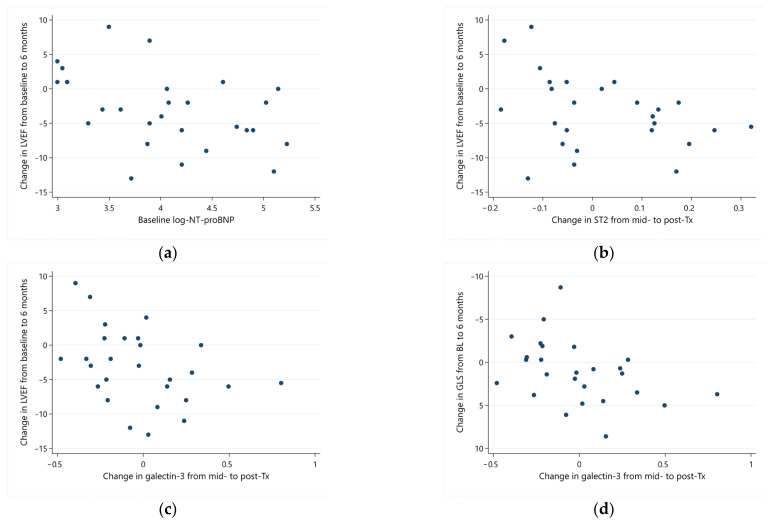
Correlation of serial biomarkers with echocardiographic parameters. (**a**) Baseline log-transformed NT-proBNP and changes in LVEF from baseline to 6 months; (**b**) changes in log ST2 (from mid- to post-therapy) and LVEF (from baseline to 6 months); (**c**) changes in log galectin-3 (mid- to post-therapy) and LVEF (from baseline to 6 months); (**d**) changes in log galectin-3 (mid- to post-therapy) and GLS (from baseline to 6 months).

Baseline NT-proBNP was the strongest predictor of LVEF response from baseline to 6 months (ρ = −0.45; *p* = 0.019). Higher baseline NT-proBNP was associated with a more pronounced drop in LVEF (Figure 3a). Post-therapy NT-proBNP values had a similar predictive value for the final LVEF response (ρ = −0.40; *p* = 0.038). Interim changes (from mid-therapy to post-therapy) in ST2 and galectin-3 also correlated with 6-month change in LVEF (ρ = −0.48; *p* = 0.012 and ρ = −0.45; *p* = 0.018, for ST2 and galectin-3, respectively), with increasing values of these biomarkers predicting an adverse response and vice versa (Figure 3b,c).

Galectin-3 showed the strongest correlation with changes in GLS. Changes in galectin-3 from baseline and mid-therapy assessments paralleled the change in GLS. The response of galectin from mid- to post-therapy correlated with the change in GLS from baseline to 6 months (ρ = 0.48; *p* = 0.014), demonstrating predictive value from a clinical standpoint, as the response of galectin-3 preceded the response of GLS (Figure 3d). A reduction in galectin-3 (negative change—leftwards in Figure 3d) was associated with improved GLS at 6 months (more negative values—upwards in Figure 3d).

### 3.6. Chemotherapy/Medication Alterations and Changes in LVEF, GLS, and Biomarkers

There were no alterations in chemotherapy regimens secondary to subclinical cardiotoxicity. Between the baseline and 6-month visits, five patients were started on a beta-blocker, two patients on an angiotensin-converting-enzyme inhibitor (ACE-I), and two on an angiotensin receptor blocker. Baseline medications or initiation/changes in cardioprotective medications did not predict changes in LVEF or GLS.

## 4. Discussion

Decisions regarding surveillance and treatment of cardiotoxicity among cancer patients receiving anthracycline chemotherapy remain largely center-specific. Most anthracycline-induced cardiotoxicity occurs within the first year of therapy and is associated with cumulative anthracycline dose and LVEF at the end of treatment [3]. Early detection and treatment of cardiotoxicity are paramount to substantial recovery of cardiac function. The 2020 guidelines from the European Society of Cardiology Cardio-Oncology Study Group and International Cardio-Oncology Society suggest utilizing baseline characteristics including history of prior cardiovascular disease, biomarkers such as troponin and NT-proBNP, demographics, cardiovascular risk factors, prior chemotherapy treatment, and lifestyle factors for the purposes of anthracycline-induced cardiotoxicity risk stratification [21]. The prospective study presented here sought to investigate the role of novel cardiac biomarkers in predicting subclinical cardiotoxicity.

In this prospective study that included 31 patients receiving anthracycline chemotherapy, LVEF was significantly reduced immediately and 6 months post-anthracycline chemotherapy. Our data suggests that even small changes in LVEF can be detected early in a patient’s clinical course. In addition to LVEF, strain imaging has become a widely used modality for the assessment of left ventricular function to detect subclinical changes. Strain reflects the deformation of the ventricular myocardium as a percentage of the initial length [19]. In our study, 8/30 (27%) and 10/26 (38%) patients developed subclinical cardiotoxicity post- and 6 months post-anthracycline therapy, respectively. We found that GLS was not significantly changed at 6 months post-anthracycline follow-up; however, there was a trend towards a decrease in GLS, suggesting that there may be significant changes at a time point beyond 6 months. Diastolic LV tissue velocities were reduced over time; however, the E/E’ ratio remained relatively stable as E wave velocities decreased too.

Many studies have noted associations between biomarkers and cardiotoxicity. The PREDICT trial found that a baseline BNP > 100 pg/mL and elevated BNP > 100 pg/mL at any time during the study were associated with cardiotoxicity [22]. Another study consisting of 323 breast cancer patients receiving anthracyclines and/or trastuzumab found that changes in NT-proBNP were associated with changes in LVEF and cancer therapy-related cardiac dysfunction [23], confirming smaller studies that illustrated persistent elevations in BNP portend worse prognosis [11,12]. Our study illustrated a correlation between a reduced LVEF and increased NT-proBNP at baseline and post-therapy. In general, while several biomarker levels rose during treatment, almost all remained within normal limits. Whether these subclinical changes have long-term clinical sequelae deserves further investigation.

Early increases in cardiac troponin I after anthracycline use may predict diastolic dysfunction [24]. Ky et al. found that interval changes in troponin I were associated with subsequent cardiotoxicity [25]. Further, this same study suggested that the frequency of biomarker monitoring for cardiotoxicity during and after anthracycline therapy should depend upon baseline risk factors for cardiovascular disease, with the highest risk patients obtaining NT-proBNP and troponin levels prior to the second, fourth, and sixth cycles of chemotherapy [25]. In our study, we were not able to draw any conclusions on troponin correlation to cardiotoxicity since all troponin levels were below the lower limit of detection.

Elevation in galectin-3 was recently correlated with the development of anthracycline-induced cardiotoxicity in breast cancer patients [17], but there remains limited data on the role of galectin-3 in the breast cancer population. In our study, changes in galectin-3 from mid-therapy to post-therapy correlated with changes in LVEF over the 6-month study period. Further, changes in galectin-3 from baseline and mid-therapy assessments paralleled the change in GLS. Further, the galectin-3 levels improved when the GLS improved at the post-therapy and 6-month post-therapy visits, suggesting a correlation between GLS and galectin-3.

ST2 has been shown to provide unique prognostic information independent of NT-proBNP [16,26]. This was a biomarker of interest in our study, and we found that ST2 did increase throughout the course of chemotherapy, correlating with the final LVEF.

Prevention of anthracycline-induced cardiotoxicity remains the subject of significant interest. Cardinale et al. demonstrated that early initiation of an HF regimen for asymptomatic patients with LVEF ≤ 45% correlated with systolic recovery. Importantly, no improvement in LVEF was observed when HF treatment was delayed by more than 6 months [27]. A meta-analysis showed several neurohormonal antagonists, including spironolactone, beta-blockers, and ACE-I, may have a cardioprotective role, though data has been mixed [28]. The OVERCOME trial illustrated there is preservation of LVEF from early initiation of beta-blocker and ACE-I therapy after anthracycline utilization in patients with normal baseline LVEF [29]. A meta-analysis of eight randomized controlled trials that studied utilization of carvedilol for primary prevention of anthracycline-induced cardiotoxicity found that, overall, there were smaller reductions in LVEF among those who were treated with carvedilol [30]. In the PRADA trial, ACE-I was associated with a modest reduction in left ventricular diastolic volume, attenuated reduction in LVEF, and preservation of GLS, though the effect size was small [31]. Conversely, other trials have failed to demonstrate a clear benefit to a cardioprotective treatment strategy in cancer patients receiving cardiotoxic agents. The CECCY trial showed no benefit of beta-blockers in preventing early LVD, though a modest reduction in diastolic dysfunction was observed [32]. Boekhout et al. found that the use of an angiotensin II receptor blocker in patients receiving anthracycline-based chemotherapy followed by trastuzumab did not prevent LVD [33]. Our study did not show a clear benefit of continuation or initiation of cardioprotective medications, though we did find that patients receiving beta-blockers had associated improvements in galectin-3 after anthracycline therapy was completed. This may ultimately translate into an improvement in GLS and LVEF in the long term based on our findings reported earlier, which illustrated a correlation between galectin-3 and GLS. Though data on early initiation of a cardioprotective strategy for anthracycline patients is modest and mixed, rationale still exists for early identification of subclinical toxicity, particularly in higher-risk patients or in patients who may require ongoing cardiotoxic chemotherapy.

This study adds to the literature on the use of biomarkers in the prediction of cardiotoxicity among breast cancer patients receiving anthracyclines, with ST2, galectin, and NT-proBNP suggesting the most promise. Here, we have utilized noninvasive testing, including biomarkers and two-dimensional or three-dimensional strain imaging, to assess cardiotoxicity at regular intervals. With the noninvasive nature of this testing, obtaining imaging and biomarkers at baseline and at various intervals early in cancer therapy may be of incremental benefit to these patients. Until further data suggests evidence of a more tailored approach, it is worthwhile to consider continuous monitoring of these patients pre-, during, and post-anthracycline therapy.

### Study Limitations

There are several limitations in our study. This study was a pilot cohort limited to 31 subjects and, therefore, lacked the power to detect significant changes in biomarker and echocardiographic parameters that may exist. In addition, this study was limited to 6 months of follow-up, whereas longer observation may have provided further insight. Due to the dynamic nature of breast cancer treatment-related issues, several patients were unable to complete all planned follow-ups due to noncardiac clinical events. Patients included in this study were primarily of Caucasian descent and, therefore, may limit the ability to extrapolate the findings to other populations. Further, many of the patients in this study did not have many cardiac risk factors, including significant coronary artery disease, thus limiting the applicability of the data to those patients who might be considered at higher risk for cardiac toxicity. Research-related restrictions due to the COVID-19 pandemic impacted patient enrollment, follow-ups, and timely data collection. Most notably, the definitions for overt and subclinical cardiac toxicity are reflective of the standard practice at the time of study design and enrollment and differ slightly from the currently accepted criteria as put forth in current guidelines [34].

## 5. Conclusions

In this single-center prospective study of women with breast cancer treated with anthracycline-based chemotherapy, we observed that early changes in cardiac biomarkers, including NT-proBNP, correlate with changes in LVEF over the course of 6 months. In addition, galectin-3 and ST2 correlate with changes in GLS in these patients. These preliminary data suggest that established and novel biomarkers can improve our ability to predict cardiotoxicity and personalize care for these patients.

A serum biomarker approach for early identification, risk stratification, and monitoring of chemotherapy-related cardiotoxicity holds promise, though challenges exist with respect to the timing of measurement, optimal assays, and whether a biomarker strategy is best used alone or as part of a more comprehensive surveillance approach.

Larger randomized controlled trials are needed to establish specific guidelines for medical therapy in both prophylaxis and treatment of anthracycline-induced cardiotoxicity [28].

## 6. Perspectives

Competency in medical knowledge: In patients who are receiving anthracycline-based chemotherapy for breast cancer, early changes in biomarkers, including NT-proBNP, correlate with changes in LVEF. In addition, galectin-3 and ST2 correlate with changes in GLS.

Translational outlook: Further studies are needed to assess the long-term correlation between biomarkers and subclinical LVD, which will ultimately enable the utilization of HF medications prior to clinically evident HF.

## Figures and Tables

**Table 1:** Baseline characteristics.

Characteristic	Value
**Demographics**	
Age	50 (46–55)
Height (cm)	165 (160–167)
Weight (kg)	73 (64–86)
BMI (kg/m^2^)	26.6 (23.7–31)
Race, %	
White	25 (80.6)
Black	2 (6.5)
Hispanic	2 (6.5)
**Comorbidities, %**	
Diabetes	2 (6.5)
Hypertension	10 (32.3)
Hyperlipidemia	7 (22.6)
**Social and family medical history**	
Smoking, %	
Never	16 (53.3)
Former (quit > 6 months)	9 (30)
Active (current or quit < 6 months)	5 (16.7)
Number of pack years	10 (7.5–20)
Alcohol abuse, %	1 (3.2)
Drug abuse, %	1 (3.2)
Family history of coronary artery disease, %	6 (19.4)
Family history of breast cancer, %	4 (12.9)
**Past drug history**	
Chemotherapy regimen, %	
Dose-dense doxorubicin and cyclophosphamide	2 (6.5)
Dose-dense doxorubicin and cyclophosphamide-paclitaxel	25 (80.6)
ddAC-T plus trastuzumab	4 (12.9)
Anthracycline dose (mg/m^2^)	60
Cyclophosphamide dose (mg/m^2^)	600
Paclitaxel (mg/m^2^)	80 (80–175)
Trastuzumab dose (mg/m^2^)	8
Chemotherapy cycles, %	
Number = 4	27 (90)
Number = 12	3 (10)
Angiotensin-converting-enzyme inhibitor %	3 (9.7)
Angiotensin receptor blocker, %	3 (9.7)
Beta-blocker, %	4 (12.9)
Metoprolol	1 (3.2)
Atenolol	1 (3.2)
Carvedilol	1 (3.2)
Statin, %	1 (3.2)
Noninsulin diabetic medication, %	1 (3.2)
Metformin	1 (3.2)
DPP-4 inhibitors (sitagliptin)	1 (3.2)
Calcium channel blocker, %	3 (9.7)
Diltiazem	2 (6.5)
Amlodipine	1 (3.2)
Hydrochlorothiazide	3 (9.7)
Triamterene	1 (3.2)
Other lipid-lowering agents, %	2 (5.26)
**Physical examination**	
Vital signs	
Systolic blood pressure (mmHg)	123 (111–140)
Diastolic blood pressure (mmHg)	77 (68–84)
Heart rate, (beats per minute bpm)	73 (68–84)
Peripheral edema. %	
None	30 (96.8)
Trace	1 (3.2)
**EKG**	
QTc (milliseconds ms)	415 (405–427)
Left anterior fascicular block, %	2 (6.7)
**Biochemistry**	
Potassium (millimole mmol/Liter L)	4.1 (4–4.2)
Glomerular filtration rate (mL/min/1.73 m^2^)	102 (96–107)
Alanine aminotransferase (international units IU/L)	16.5 (12.5–25.5)
Aspartate aminotransferase (IU/L)	19.5 (16–26)
Total cholesterol (mg/dL)	203 (181.5–227)
Low-density lipoprotein (mg/dL)	110 (86–123)
High-density lipoprotein (mg/dL)	72 (62–81)
White blood cells (K/uL)	6 (5.4–7.5)
Hemoglobin (g/dL)	12.9 (12.3–13.7)
Platelets (K/uL)	253 (215–276)
**Tumor findings, %**	
Tumor classification	
T1	8 (25.8)
T2	15 (48.4)
T3	7 (22.6)
Lymph node	
Positive	25 (80.6)
Negative	5 (16.1)
Node classification	
N0	7 (23.3)
N1	18 (60)
N2	4 (13.3)
N3	1 (3.3)
Human epidermal growth factor receptor 2 +	6 (19.4)
Estrogen receptor +	23 (74.2)
Progesterone receptor +	21 (67.7)
Ki-67 protein +	24 (100)

ddAC-T = dose-dense doxorubicin and cyclophosphamide-paclitaxel; + = positive.

**Table 2:** Summary of follow-up assessments.

	ECG	Echocardiography	Biomarkers
**Baseline**	31	31	31
**Mid-therapy**	N/A	N/A	30
**Post-therapy**	29	31	30
**Six months**	27	27	27

**Table 3:** Echocardiographic findings at baseline, immediately post-anthracycline therapy completion, and 6 months after anthracycline therapy completion.

Characteristic	Baseline (N = 31)	Post-Tx (N = 28)	6-Month Post (N = 29)	*p* Value *
Ejection fraction (%)	63.0 (60.0, 65.0)	60.0 (58.0, 63.0)	60.0 (56.0, 63.0)	0.005
LVIDd (cm)	4.6 (4.3, 4.9)	4.6 (4.5, 4.8)	4.6 (4.25, 4.77)	0.53
LV FS (%)	33.7 (28.4, 36.7)	34.8 (30.9, 36.3)	32.6 (27.6, 35.4)	0.12
LV EDV (mL)	89 (82, 103)	91 (66, 106)	87 (77, 101)	0.18
LV ESV (mL)	33 (30, 40)	35 (26, 42)	37 (28, 43)	0.76
LV SV (mL)	56.1 (41.0, 68.3)	49.1 (40.2, 57.9)	49.2 (41.1, 55.9)	0.018
LA diameter (cm)	3.1 (2.8, 3.6)	3.3 (2.9, 3.7)	3.3 (2.8, 3.5)	0.73
LA area (cm^2^)	16.5 (13.1, 19.1)	16.2 (14.5, 18.4)	14.0 (12.2, 16.4)	0.014
LA volume (mL)	42.0 (32.0, 56.2)	43.9 (39.8, 54.6)	38.4 (29.4, 46.0)	0.046
LV SI (mL/m^2^)	32.7 (23.9, 35.8)	26.3 (22.4, 32.8)	27.5 (22.9, 30.8)	0.016
LA volume index (mL/m^2^)	25.6 (18.5, 29.9)	26.2 (22.3, 28.9)	22.0 (16.6, 26.4)	0.022
E velocity (m/s)	0.8 (0.7, 0.9)	0.7 (0.7, 0.8)	0.7 (0.6, 0.8)	0.005
A velocity (m/s)	0.7 (0.5, 0.9)	0.7 (0.5, 0.9)	0.7 (0.5, 0.8)	0.52
E/A	1.1 (0.9, 1.5)	1.0 (0.8, 1.3)	1.2 (0.8, 1.4)	0.027
Deceleration (ms)	191.5 (170.0, 214.0)	172.5 (162.5, 191.0)	170.0 (154.0, 187.5)	0.088
Septal E’ (m/s)	0.09 (0.07, 0.12)	0.09 (0.08, 0.10)	0.08 (0.07, 0.09)	0.007
Lateral E’ (m/s)	0.12 (0.09, 0.14)	0.12 (0.09, 0.13)	0.11 (0.08, 0.12)	0.001
Average E’ (m/s)	0.10 (0.08, 0.13)	0.10 (0.09, 0.12)	0.09 (0.08, 0.10)	0.001
Average E/E’	7.6 (6.4, 9.4)	7.1 (6.0, 8.5)	7.5 (6.7, 8.4)	0.52
LA pressure (mmHg)	11.3 (9.8, 13.6)	10.7 (9.3, 12.5)	11.2 (10.1, 12.3)	0.51
TR velocity (m/s)	2.0 (1.9, 2.3)	2.3 (2.1, 2.5)	2.2 (2.0, 2.3)	0.22
Estimate PASP (mmHg)	20.8 (17.0, 24.9)	23.8 (20.7, 27.2)	22.0 (18.8, 24.6)	0.21
Global longitudinal strain (%)	−19.6 (−21.0, −17.7)	−19.0 (−17.4, −21.3)	−18.5 (−16.5 −21.2)	0.32
Diastolic dysfunction, N (%)				0.11
Normal	26 (83.9)	22 (71.0)	18 (69.0)	
Mild	5 (16.1)	9 (29.0)	7 (27.0)	
Impaired	---	---	1 (4.0)	

* Joint contrast for post-therapy and 6-month post-therapy values vs. baseline. Abbreviations: LVIDd = left ventricular internal diameter in diastole; LV FS = left ventricular fractional shortening; LV EDV = left ventricular end-diastolic volume; LV ESV = left ventricular end-systolic volume; LV SV = left ventricular stroke volume, LV SI = left ventricular sphericity index; LA = left atrium; TR = tricuspid regurgitation; PASP = pulmonary artery systolic pressure. Values are median (25th, 75th percentile).

**Table 4:** Biomarker findings at baseline, during ATC therapy, immediately post-ATC therapy completion, and 6 months after ATC therapy completion.

Biomarker	Baseline	Mid-Tx	Post-Tx	6-Month Post	*p* Value *
CRP Level	0.3 (0.0, 0.8)	-	-	0.25 (0.00, 0.65)	0.83
NT Pro-BNP	58.5 (37.0, 100.0)	76.5 (39.0, 163.0)	99.0 (75.0, 141.0)	71.0 (38.0, 151.0)	<0.001
ST-2	21.3 (15.6, 24.9)	23.3 (16.3, 33.8)	24.1 (18.6, 30.8)	22.2 (17.8, 26.4)	0.10
Galectin-3	16.3 (14.2, 18.5)	17.5 (15.5, 19.6)	16.3 (14.6, 18.8)	15.6 (13.8, 18.0)	0.038

* Joint test for change vs. baseline value. Values are median (25th, 75th percentile). Abbreviations: CRP = C-reactive protein; NT Pro-BNP = N-terminal pro-brain natriuretic peptide; ST-2 = suppression of tumorigenicity 2.

## Data Availability

The data presented in this study are available on request from the corresponding author. The data are not publicly available due to privacy and ethical restrictions.
